# Strategies for the induction of anti-inflammatory mesenchymal stem cells and their application in the treatment of immune-related nephropathy

**DOI:** 10.3389/fmed.2022.891065

**Published:** 2022-08-19

**Authors:** Cheng Zhou, Xue-Yuan Bai

**Affiliations:** Department of Nephrology, The First Medical Center, Chinese PLA General Hospital, State Key Laboratory of Kidney Diseases, National Clinical Research Center for Kidney Diseases, Beijing Key Laboratory of Kidney Diseases, Beijing, China

**Keywords:** anti-inflammatory, induction, treatment, immune-related nephrology, MSC

## Abstract

Mesenchymal stem cells (MSCs) have potent immunomodulatory functions. Animal studies and clinical trials have demonstrated that MSCs can inhibit immune/inflammatory response in tissues and have good therapeutic effects on a variety of immune-related diseases. However, MSCs currently used for treatment are a mixed, undefined, and heterogeneous cell population, resulting in inconsistent clinical treatment effects. MSCs have dual pro-inflammatory/anti-inflammatory regulatory functions in different environments. In different microenvironments, the immunomodulatory function of MSCs has plasticity; therefore, MSCs can transform into pro-inflammatory MSC1 or anti-inflammatory MSC2 phenotypes. There is an urgent need to elucidate the molecular mechanism that induces the phenotypic transition of MSCs to pro-inflammatory or anti-inflammatory MSCs and to develop technical strategies that can induce the transformation of MSCs to the anti-inflammatory MSC2 phenotype to provide a theoretical basis for the future clinical use of MSCs in the treatment of immune-related nephropathy. In this paper, we summarize the relevant strategies and mechanisms for inducing the transformation of MSCs into the anti-inflammatory MSC2 phenotype and enhancing the immunosuppressive function of MSCs.

## Overview of mesenchymal stem cells

Stem cells can be divided into three main categories: ESC (embryonic stem cells), iPSC (induced pluripotent stem cells) and MSC. ESC represents the inner cell mass of the blastocyst and possesses a pluripotent differentiation capacity. However, undifferentiated ESC can form teratomas and malignant teratocarcinoma *in vivo*, which is of high risk for direct clinical treatment. Moreover, ESC has the ability to form all three layers, so its use may raise clinical ethical issues. In the process of cell subculture, the iPSC could transfer more passages than ESC. The passage ability of iPSC was higher than ESC. However, clinical application of iPSC also has the risk of causing teratomas and malignant teratocarcinoma. Compared with ESC and iPSC, MSCs are easier to isolate and preserve, have a lower risk of clinical tumorigenesis, and do not raise ethical issues (1).

MSCs have immunomodulatory properties and tissue regeneration capabilities (2, 3). MSCs are present in various tissues, including bone marrow, adipose tissue, amniotic membrane and amniotic fluid, placenta and fetal tissue, umbilical cord tissue, endometrium, blood and synovial fluid. Because MSCs have the potential to differentiate into osteoblasts, chondrocytes, adipocytes and other mesoderms and have immunoregulatory functions, they have great application prospects in regenerative medicine research and the treatment of immune diseases (4, 5). However, MSCs can exhibit different morphological and physiological characteristics in different culture environments, and the currently prepared MSCs are heterogeneous mixed cell populations (3, 6). The International Society for Cellular Therapy has provided the minimum necessary criteria for a cell to be defined as an MSC: CD105, CD73, and CD90 positive and CD45, CD34, CD14 or CD11b, CD79a or CD19 and HLA-DR negative, with the ability to differentiate into osteoblasts, adipocytes and chondrocytes *in vitro* (7, 8). Therefore, the definition of the pro-inflammatory and anti-inflammatory subpopulations of MSCs still needs to be explored in depth.

## Immunomodulatory effects of MSCs

In recent years, many preclinical and clinical studies have conducted extensive investigations on the therapeutic potential and safety of MSCs in terms of immune regulation and the regenerative repair of diseases (9–12). The anti-inflammatory and immunomodulatory functions of MSCs have potent therapeutic effects on immunity/inflammation and autoimmune diseases (11, 12). MSCs have a wide range of immunomodulatory capabilities and can affect adaptive and innate immune responses. Therefore, MSCs are also considered a type of effective immune regulatory cell and are one of the best candidate cells for the treatment of inflammatory and autoimmune diseases. Recent studies have also shown that MSCs can interact with components in the body's immune microenvironment such as Toll-like receptors (TLRs) and cytokines. Through these interactions, MSCs can exhibit anti-inflammatory or pro-inflammatory effects (13–15). This dual immunomodulatory property of MSCs may be the reason for the differences between the results of preclinical and clinical studies and the significant difference in the therapeutic effects of MSCs among different studies. Therefore, the immunomodulatory properties of MSCs need to be studied in depth in order to optimize MSC-based treatment regimens.

## Immunophenotyping of MSCs

Similar to macrophages, which can polarize into pro-inflammatory M1 or anti-inflammatory M2 phenotypes, MSCs can exhibit two phenotypes, pro-inflammatory MSC1 or anti-inflammatory MSC2, as a result of different inflammatory environments (16, 17). The pro-inflammatory MSC1 phenotype seems to help establish an appropriate early inflammatory response, while the anti-inflammatory MSC2 phenotype helps to inhibit the inflammatory response. MSC1 can secrete more pro-inflammatory cytokines, such as interleukin 6 (IL-6) and IL-8, to promote the activation of T cells, and MSC2 can secrete more anti-inflammatory cytokines, such as indoleamine-2,3-dioxygenase (IDO), IL-10, and prostaglandin E2 (PGE2), to inhibit the activation of T cells. Elucidating the molecular mechanisms that regulate the phenotypic transition of MSC1 and MSC2 and adopting specific methods and strategies to enhance the immunosuppressive ability of MSCs will have important significance for the future clinical application of MSCs in the treatment of immune/inflammatory diseases.

Although MSCs were first reported to be derived from bone marrow, a number of studies have reported similar cell types in a wide range of tissues, e.g., umbilical cord blood, placenta, adipose tissue, amniotic fluid, dental tissue, skin, hair follicles and tonsils. At present, most of MSCs used are bone marrow (BM-MSCs), umbilical cord (UC-MSCs), umbilical cord blood (CB-MSCs), placenta (P-MSCs) and adipose tissue (A-MSCs). Studies have found that MSCs from different sources do have different functions and characteristics, and therefore different therapeutic effects. P-MSCs showed a slight increase in growth, BM- and A-MSCs possess the highest capacity for self-renewal and differentiation potential in multiple lineages, whereas P-MSCs have the least functionality as stem cells of those which were tested. UC-MSCs were shown to express superior clonogenicity, migration, and paracrine capacities *in vitro*, as well as less senescence when compared with BM-MSCs. At present, UC-MSCs, BM-MSCs and A-MSCs are mainly used in inducing MSC2 (18).

## Strategies to induce the production of MSCs with different phenotypes

**Figure 1:**
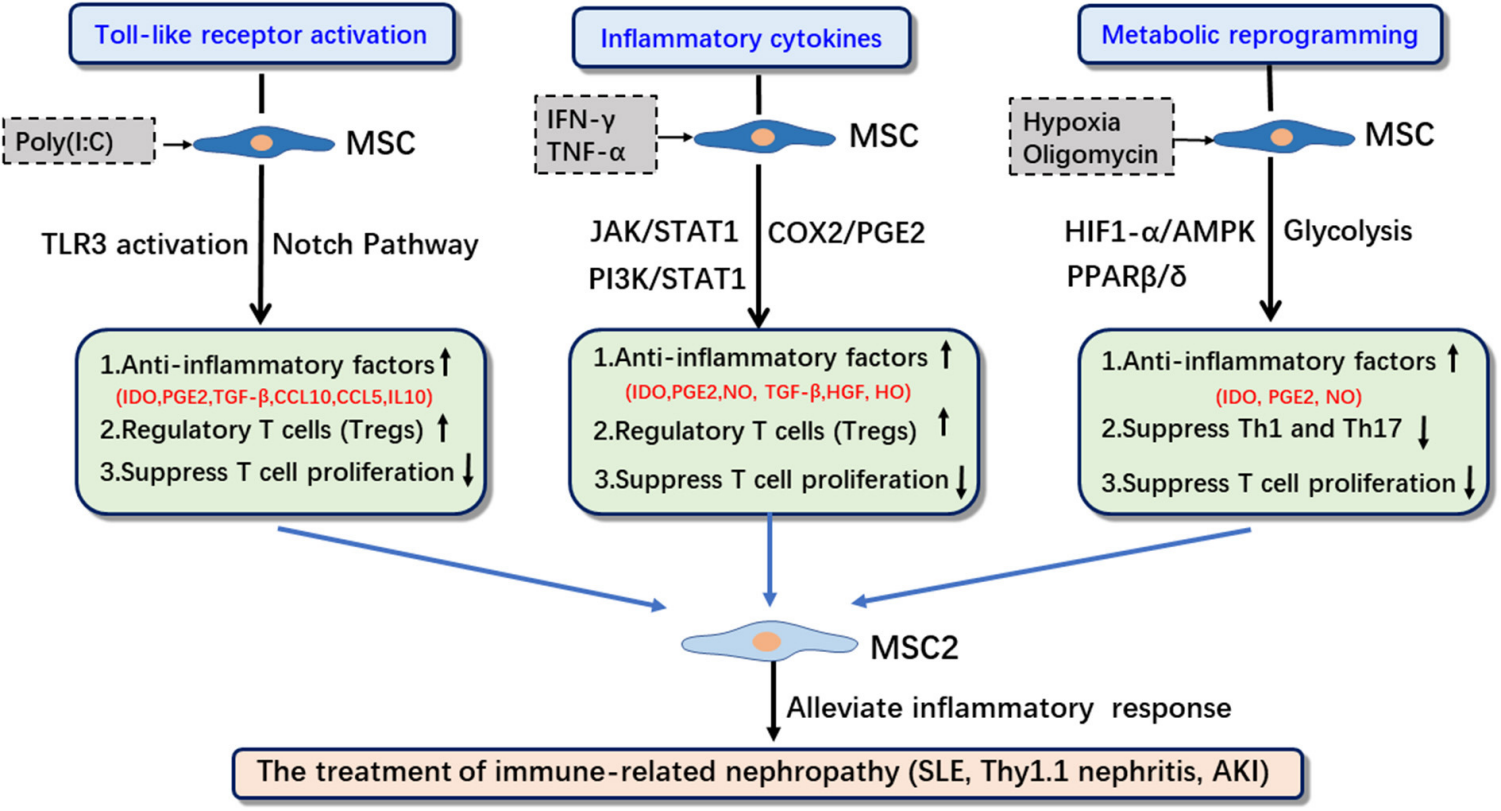
The induction strategies, related signaling pathways and applications of MSC2. IDO, indoleamine 2,3-dioxygenase; PGE2, prostaglandin E2; HO, heme oxygenase-1; SLE, systemic lupus erythematosus; IRI, ischemia reperfusion injury; Thy1.1, Thy-1.1 nephritis.

Studies have found that the activation of TLRs, the inflammatory cytokine microenvironment and the activation of glycolysis in the metabolic reprogramming play important roles in the immunosuppressive functional transformation of MSCs (Figure 1). MSCs can be activated by pathogen-associated molecular patterns (PAMPs). PAMPs activate MSCs by binding to pattern recognition receptors (PRRs) on MSCs (19, 20). MSCs express multiple TLRs; furthermore, their ability to migrate, invade, and secrete immunomodulatory factors is also strongly influenced by specific TLR agonists. The activation of different types of TLRs can drive the transformation of MSCs into the MSC1 or MSC2 phenotype. Pro-inflammatory cytokines such as IFN-γ, TNF, and IL-1β can enhance the immunosuppressive function of MSCs (21). A pro-inflammatory environment, e.g., the presence of INF-γ and IL-6, can induce MSCs to transform into anti-inflammatory MSC2 and secrete the anti-inflammatory factor PGE2 (22). Studies have also confirmed that the metabolic changes in MSCs are related to their immunomodulatory functions (23). The metabolic transformation of MSCs to aerobic glycolysis can significantly regulate the immunomodulatory properties of MSCs by regulating the production of IDO, thereby enhancing T cell inhibition and anti-inflammatory effects (24).

In this paper, we will focus on the role of TLR activation, inflammatory cytokines, and metabolic reprogramming, as well as the correlation among them, in enhancing the immunosuppressive function of MSCs to identify optimal strategies and methods to promote the transformation of MSCs into MSC2 (Figure 1, Table 1).

**Table 1:** The induction strategies of MSC2 and its applications in clinical or pre-clinical studies.

**Authors**	**Clinical or pre-** **clinical studies**	**Stimulus**	**Stimulus duration of the**	**Stimulus dose**	**Follow** **up period**	**Main goal and outcome**
Jang et al. (25)	SLE(LN) (mouse)	metformin	72 h	1 × 10^6^ Ad-MSCs	16 weeks	1. Metformin promoted immunoregulatory effect of Ad-MSCs by enhancing STAT1 expression 2. Metformin-treated Ad-MSCs inhibited CD4^−^, CD8^−^ T-cell expansion and Th17/Treg cell ratio
Ishiuchi et al. (26)	Renal fibrosis (mouse)	serum-free medium+ 1% O2	24 h	5 × 10^5^ BM-MSC	7 or 21 days	1. Hypo-SF-MSCs ameliorated renal fibrosis. 2. Hypo-SF-MSCs attenuated infiltration of inflammatory cells 3. Hypo-SF-MSCs inhibited TGF-β/Smad signaling
Kanai et al. (27)	Renal fibrosis (mouse)	IFN-γ	24 h	5 × 10^5^ BM-MSC	7 or 21 days	1. IFN-γ-treated MSCs reduced infiltration of inflammatory cells cells and ameliorated interstitial fibrosis 2. IFN-γ-treated MSCs increased secretion of prostaglandin E2
Bai et al. (28)	IRI (mouse)	IL-17A	48 h	1 × 10^6^ BM-MSC	24 or 72 h	1. IL-17A-pretreated MSCs increased the percentages of Foxp3+ Tregs 2. IL-17A-pretreated MSC therapy lowered serum IL-6 TNF-α, and IFN-γ levels 3. IL-17A upregulated COX-2 expression and increased PGE2 production
Xu et al. (29)	SLE (mouse)	IL-37	overexpressing IL-37	1 × 10^6^ BM-MSC	7 weeks	1. MSCs-IL37 suppress B Cells, increas CD4+Foxp3+ cells in PBMCs of MRL/lpr mice 2. MSCs-IL37 had elevated production of IL-37 after transplantation
Deng et al. (30)	Thy1.1 (rat)	Chlorzoxazone (CZ)	24 h	-	3 days	1. CZ-treated MSCs alleviate infiltration of inflammatory cells 2. CZ-treated MSCs inhibit T cells activation and proliferation
Waterman et al. (31)	Painful diabetic peripheral neuropathy (rat)	Poly(I:C)	1 h	1 × 10^6^ BM-MSC	40 days	Mice treated with MSC2 decreased serum Pro-inflammatory cytokines
Kurte et al. (32)	Experimental Autoimmune Encephalomyelitis (rat)	LPS	48 h	BM-MSC	21 days	MSCs-LPS inhibit T cell proliferation, improve therapeutic effect and increase Treg
Fuenzalida et al. (33)	DSS induced colitis (mouse)	poly(I:C)	1 h	1 × 10^6^ UC-MSCs	11 days	UCMSCs pre-conditioned with poly(I:C) ameliorates DSS-induced coliti
Yu et al. (34)	IRI (mouse)	Cobalt chloride (CoCl2)	24 h	—	72 h	CoCl2-treated MSCs have greater migration and longer retention time; Reduced kidney injury
Contreras-Lopez et al. (35)	DTH and GVHD (mouse)	ATP synthase (oligomycin)	24 h	1 × 10^6^ MSC	7 days	MSC glycolytic reprogramming increased their therapeutic benefit
Contreras-Lopez et al. (36)	DTH (mouse)	HIF1α	HIF1α knock-down	—	24 h	HIF1αexpression is critical for the therapeutic potential of MSC by reducing pro-inflammatory Th1 and Th17 cells
He et al. (37)	Clinical rheumatoid arthritis (RA) (human)	IFN-γ	(hUC-MSCs) combined with human IFN-γ	1 × 10^6^ cells/kg	1–48 weeks	MSC plus IFN-γ combination can improve clinical efficacy of MSC-based therapy
Daneshmandi et al. (38)	Type 1 diabetes (mouse)	TGF-β	TGF-βtransduced MSCs	5 × 10^5^ BM-MSC	5 weeks	Engineered TGF-β/MSCs alleviate T1D by regulation of adverse immune responses

IRI, ischemia reperfusion acute kidney injury; DTH, the Delayed-Type Hypersensitivity; GVHD, the Graph vs. Host Disease.

### TLR activation

The TLR family includes TLR1-10 (39). Most of the risk signals that trigger TLRs are released after tissue damage. Exogenous risk signals are usually released after microbial infection. The most common signals are endotoxin or lipopolysaccharide (LPS); endogenous risk signals are mainly intracellular components, such as heat shock proteins (HSP) or RNA, that are released into the circulation from abnormal or injured cells. Under normal circumstances, these risk signals can activate TLRs on immune cells to initiate inflammatory responses (40). Studies have shown that TLR2 activation may be involved in the differentiation, migration and proliferation of MSCs and that TLR3 and TLR4 may be involved in the remodeling of MSC immunoregulation (41).

MSCs can express a variety of TLRs (such as TLR3 and TLR4), and their migration, invasion, and secretion of immune regulatory factors are all affected by specific TLR agonists (42). MSCs can polarize into two different phenotypes *via* TLR downstream signaling pathways. Two different phenotypes of MSCs have been defined based on TLR activation: MSC1 (pro-inflammatory phenotype) and MSC2 (anti-inflammatory phenotype) (41). TLR3 activation can enhance the secretion of most anti-inflammatory cytokines, such as IDO, IL-10, PGE2, and IL-4, and can inhibit the activation of T cells; that is, TLR3 is associated with the MSC2 phenotype. TLR4 activation leads to the secretion of more pro-inflammatory cytokines, such as IL-6 and IL-8, which can promote the activation of T cells. TLR4 is associated with the MSC1 phenotype (31). It was found that treatment of MSCs with a TLR4 agonist (LPS) for 1 h promoted the transformation of MSCs into the MSC1 pro-inflammatory phenotype and that the treatment of MSCs with a TLR3 agonist (poly(I:C)) for 1 h promoted the transformation of MSCs into the MSC2 anti-inflammatory phenotype (41). In addition, the duration of LPS treatment can also change the expression of TLR receptors, thereby affecting the transformation of MSCs into the pro-inflammatory or anti-inflammatory phenotype (32). However, the molecular mechanisms underlying how activations of TLR3 and TLR4 affect the pro-inflammatory and anti-inflammatory phenotypic transformation of MSCs have not been elucidated. TLR signaling pathways are strictly regulated by a variety of mechanisms. For example, TLR4 can activate two signaling pathways (dependent or independent of MyD88) (43). The activation of different pathways may have different effects on the immunosuppressive ability of MSCs. Currently, most studies have not specifically elucidated which pathway is used to regulate the polarization of MSCs into the pro-inflammatory or anti-inflammatory phenotype after TLR3 or TLR4 activation.

### Activation of inflammatory cytokines

MSCs without treatment have the weakest immunosuppressive effect; the anti-inflammatory phenotype of MSCs must be activated by exposure to a specific environment (such as an inflammatory microenvironment) (44, 45). The immunosuppressive effect of MSCs requires the “licensing” of inflammatory factors. In an inflammatory environment (such as high concentrations of TNF-α and IFN-γ), MSCs are activated and inhibit T cell proliferation through the secretion of soluble factors [including IDO, PGE2, NO, TGF-β, HGF, and heme oxygenase (HO)], exhibiting an immunosuppressive phenotype (MSC2) (16). In the absence of an inflammatory environment (low concentrations of TNF-α and IFN-γ), MSCs may exhibit a pro-inflammatory phenotype (MSC1) and enhance T cell responses by secreting chemokines (e.g., MIP-1α and MIP-1β, RANTES, CXCL9, and CXCL10) to recruit lymphocytes to sites of inflammation (46, 47). When displaying the MSC1 phenotype, the levels of immunosuppressive mediators such as IDO and NO are low.

Among the inflammatory cytokines, the pro-inflammatory cytokine IFN-γ is the most studied factor that initiates the immunosuppressive capacity of MSCs (44, 48). TNF-α, IL-1α, IL-1β, IL-10, IL-17, and TGF-β can all have similar functions as IFN-γ regarding the initiation of the anti-inflammatory function of MSCs (46, 49–51). Studies have confirmed that inflammatory cytokines such as IFN-γ can induce the transformation of MSCs into the MSC2 anti-inflammatory phenotype. In a study of cytokines and TLRs in the anti-inflammatory phenotype transformation of MSCs, high concentrations of IL-17A activated TLR3 and promoted the anti-inflammatory phenotype transformation of MSCs into MSC2, and low concentrations of IL-17A activated TLR4 and promoted the pro-inflammatory phenotype transformation of MSCs into MSC1 (52). Therefore, the relationship between different pro-inflammatory cytokines and TLRs still needs to be further studied.

Elucidating the key signaling pathway components involved in the cytokine regulation of MSC immunophenotypic transformation will have important significance for the treatment of immune diseases. A key feature of MSC2 is their ability to respond to pro-inflammatory cytokines (IFN-γ) and release IDO, a key immunosuppressive molecule produced by human MSCs. The major signaling pathways activated by IFN-γ involve the Janus kinase (JAK) and signal transducer and activator of transcription (STAT) pathways (53). The response of MSCs to IFN-γ may involve the activation of different subtypes of STAT. IFN-γ can also activate phosphoinositide 3-kinase (PI3K) to induce IDO production by MSCs (54, 55). This process relies on the interaction between the PI3Kα and STAT1 pathways. STAT1 overexpression or PI3Kα pathway activation can induce the phenotypic transformation of MSCs into MSC2, significantly enhance IFN-γ-mediated IDO production, and enhance the inhibitory effect of MSCs on T cells (56). In addition to IDO, which is an immunosuppressive molecule in MSCs, PGE2 is also an immunosuppressive molecule secreted by MSCs. Bai's study found that IL-17A upregulated COX-2 expression and increased the production of PGE2, thereby enhancing the inhibitory effect of MSCs (28).

In addition to activating related signaling pathways to enhance their anti-inflammatory function, inflammatory cytokines can also have a synergistic effect with TLRs.

### Changes in metabolic reprogramming

Metabolism can significantly affect the fate of stem cells (57, 58). Studies have found that metabolic stress and metabolic reprogramming are involved in the immunomodulatory function of MSCs. Studies have shown that the changes in energy metabolism pathways play key roles in regulating the immunosuppressive activity of MSCs (23). Under normoxic conditions, MSCs produce ATP through glycolysis and oxidative phosphorylation (OXPHOS). Under hypoxic conditions, MSCs mainly produce energy through glycolysis. The metabolism of undifferentiated MSCs during the proliferation process mainly relies on glycolysis, and the metabolism of MSCs mainly relies on mitochondrial OXPHOS during the differentiation process (59, 60). MSCs are usually in a hypoxic physiological environment (such as bone marrow) in the body. However, MSCs need to be cultured and expanded *in vitro* for clinical applications, and this process promotes their metabolic reprogramming to OXPHOS, thereby reducing their treatment effect (61, 62). In contrast, MSCs cultured in an inflammatory microenvironment have a tendency to induce the transition to their glycolytic pathway and can enhance their immunomodulatory potential (24, 61). Changes in the metabolic pathways of MSCs caused by different culture and stimulation conditions have a direct impact on the characteristics of MSCs (including proliferation, senescence, differentiation, and immunosuppression). Glycolysis significantly affects the immunomodulatory properties of MSCs by regulating IDO activity (63). However, the role of MSC metabolic reprogramming in the therapeutic properties of MSCs and the key molecular mechanisms of glycolysis in MSC immunoregulation still need to be further elucidated.

Inflammation can enhance the immunosuppressive properties of MSCs and induce their glycolytic reprogramming. Pro-inflammatory cytokines (especially TNF-α and IFN-γ) can activate MSCs and trigger the release of MSCs with immunosuppressive potential (35). By comparing the metabolic activity of MSCs under basal culture conditions and the metabolic activities of MSCs incubated with TNF-α and IFN-γ for 24 h, it was found that pro-inflammatory cytokines activated MSCs, significantly reduced the basal and maximum oxygen consumption rate (OCR) and the spare respiratory capacity (SRC), and increased the extracellular acidification rate (EACR) in MSC supernatant, i.e., induced a transition to aerobic glycolysis in MSCs. Studies have also found that the TNF-α- and IFN-γ-induced glycolysis switches are associated with increased lactate output and glycolytic enzyme expression. The metabolic pathways of MSCs also affect the anti-inflammatory function of MSCs. Oligomycin (inhibition of OXPHOS) or 2DG (inhibition of glycolysis) can modify the metabolic activity of MSCs by inducing metabolic switches, thereby regulating the production of immunosuppressive mediators. After MSCs are treated with oligomycin, the metabolic mode of MSCs is converted to glycolysis-based, and the immunosuppressive ability of MSCs is enhanced. In contrast, 2DG weakens the immunosuppressive ability of MSCs.

The signaling pathways through which MSCs regulate their immunosuppressive functions *via* metabolic reprogramming include AMPK, HIF1-α, and PPARβ/δ (35, 36, 64). AMPK plays an important role in cellular metabolism as a cellular energy sensor and a master controller for the adaptive response to changes in metabolic demand (65). Studies have shown that enhancement of the glycolytic pathway during the transformation of MSCs into MSC2 is related to the upregulation of AMPK expression. AMPK can enhance the glycolytic pathway in MSCs and thus enhance the immunosuppressive activity of MSCs. Studies have found that oligomycin and pro-inflammatory cytokines increase the immunosuppressive properties of MSCs by activating the AMPK signaling pathway. Activation of oligomycin-treated MSCs by TNF-α and IFN-γ can further increase the expression level of PD-L1 and the production of other immunosuppressive mediators (such as PGE2) to enhance their anti-inflammatory phenotype. In addition, the activation of MSCs by pro-inflammatory cytokines can enhance the production of reactive oxygen species (ROS), induce the expression of HIF1-α, and change the metabolic mode of MSCs to the glycolytic pathway. HIF1-α knockdown in MSCs can reduce the expression of various inflammatory mediators in MSCs.

PPARβ/δ, a member of the PPAR family, is highly expressed in skeletal muscle and is a key regulator of fatty acid oxidation and glucose uptake (66). PPARβ/δ knockout or knockdown promotes the transformation of MSCs into glycolysis and enhances their ability to inhibit the proliferation of Th1 and Th17 cells. This finding indicates that PPARβ/δ is a key switch related to MSC immunomodulatory function. The inhibition of PPARβ/δ expression can promote the transformation of MSC metabolic reprogramming into glycolysis, thereby enhancing the immunosuppressive ability of MSCs (64).

## Application of anti-inflammatory MSC2 in the treatment of immune-related nephropathy

Inflammation plays a vital role in kidney diseases. Therefore, improving the ability of MSCs to control the inflammatory progression of kidney injury tissues is a focus of research on the application of MSCs in immune-related nephropathy. Although MSCs currently generate good results related to the treatment of immune-related nephropathy (such as systemic lupus erythematosus nephritis, Thy-1 nephritis, and renal ischemia–reperfusion, etc.), there are also studies that indicate that the efficacy of MSCs in the treatment of immune-related nephropathy is inconsistent. To address the controversy regarding the therapeutic effects of MSCs, many studies have found that inducing the transformation of MSCs into anti-inflammatory MSCs can improve the therapeutic effects.

Changes in the metabolic pattern of MSCs can affect the changes in their immunosuppressive function. Jang et al. found that in a systemic lupus erythematosus nephritis model, metformin enhanced the immunomodulatory potential of adipose-derived MSCs through STAT1, thereby improving the progression of lupus nephritis (25). Metformin directly activated AMPK to promote the expression of a series of energy metabolism-related genes, i.e., metformin induced glycolysis by activating AMPK, thereby changing cellular metabolic reprogramming (67). In addition, Ishiuchi's study found that serum-free culture and hypoxic pretreatment synergistically enhanced the therapeutic effects of MSCs on renal fibrosis and that hypoxia transformed cellular metabolism into glycolysis. Therefore, it is speculated that the production of anti-inflammatory MSCs can be induced by regulating the metabolic reprogramming of MSCs (26).

Studies have found that many inflammatory factors (such as IFN-γ, TNF-α, and IL-17A) can induce the production of anti-inflammatory MSCs. Kanai found that IFN-γ preconditioning enhanced the anti-fibrosis ability of MSCs in rats with ischemia–reperfusion injury (IRI) and unilateral ureteral obstruction (27). Bai found that IL-17A pretreatment of bone marrow MSCs improved their immunosuppressive ability and increased the percentage of Treg through the COX-2/PGE2 pathway, thereby enhancing the efficacy of MSCs in IRI-induced acute kidney injury (AKI) mice (28). Xu et al. found that IL-37 overexpression in MSCs improved the immunosuppressive effects of MSCs in systemic lupus erythematosus (29). In addition, some small molecule compounds can also induce the production of anti-inflammatory MSCs. In a study by Deng, a small molecule compound, chlorzoxazone (CZ), was screened to induce the formation of an anti-inflammatory MSC2 phenotype in MSCs; CZ enhanced the immunosuppressive ability of MSCs and more effectively reduced the inflammatory infiltration of renal tissues and glomerular fibrin-like necrosis in Thy-1 nephritis, thereby improving renal function (30) (Table 1).

Mesenchymal stem cells (MSCs) have been used to treat various immune-related diseases due to their immunomodulatory function. MSCs can regulate the progression of immune inflammation by secreting anti-inflammatory cytokines (such as IDO, PEG2, IL-10, etc.). However, mesenchymal stem cells currently used for the treatment is a mixed, undefined, heterogeneous population of cells, resulting in inconsistent clinical outcomes. MSCs can be transformed into pro-inflammatory MSC1 or anti-inflammatory MSC2 phenotype. In most immune-related diseases, the enhanced transformation from MSC into MSC2 is required to better inhibit immune inflammation. In the newly added (Table 1), we summarize how to promote the transformation of MSC into the anti-inflammatory phenotype MSC2 and its application in immune-related diseases. The anti-inflammatory function of MSC can be increased by cytokines (IFN-γ, TGF-β, IL-17A), metabolic reprogramming (Metformin, hypoxia) and TLR3 excitation (Poly(I:C)). The MSCs with the increased anti-inflammatory function mainly secretes more anti-inflammatory factors (IDO, PGE2) to inhibit the proliferation of T cells, reduce the infiltration of inflammatory cell, regulate the differentiation of T cells to reduce inflammation (such as ratio of Th1 and Th17 decreases, while Treg increases).

At present, MSCs have been widely reported in the treatment of renal diseases, while MSC2 has rarely been studied in the treatment of immune-related nephropathy. However, in other diseases, many studies have confirmed that the therapeutic effect of MSC2 is stronger than untreated MSC (31, 33–38) (Table 1).

## Summary

Studies on the immunomodulatory function of MSCs mainly focus on three aspects: 1. the association between the activation of different TLRs in MSCs and the MSC1/MSC2 phenotypes; 2. the effects of inflammation and related pathways on the anti-inflammatory effects of MSCs; and 3. the role of MSC metabolic reprogramming in the induction of MSC anti-inflammatory functions. Recent studies have confirmed that the above three aspects can all affect the pro-inflammatory and anti-inflammatory phenotypes of MSCs. However, whether the three are independent or partially dependent on each other or whether there is a unified pathway connecting the three remains to be studied. There may be a synergistic effect among the three factors; the effect of the combination may be higher than that of a single factor, and different combinations of each may be an effective strategy to enhance the immunosuppressive function of MSCs. Regulation of the key components of the inflammation-induced MSC2 pathway and metabolic reprogramming-induced MSC2 pathway, for example the PI3Kα and STAT1 pathways in the former and AMPK, HIF1-α, and PPARβ in the latter, can also enhance the immunosuppressive effect of MSCs.

The controversy regarding the efficacy of clinical stem cell application is largely due to the inconsistent definition of MSCs. Currently, the International Society for Cellular Therapy only provides the minimum criteria for a cell to be defined as an MSC. Similar to macrophages, MSCs have heterogeneity and plasticity, i.e., MSC1 (pro-inflammatory) and MSC2 (anti-inflammatory) phenotypes. Therefore, the minimum criteria can not accurately define MSCs. Studies on specific surface markers or morphological shape of MSC2 have just started, and there are no universally recognized specific surface markers on MSC2. To stably enhance the immunosuppressive function of MSCs, the relationship among the three phenotypes requires further clarification, and better methods and strategies for defining the MSC1/MSC2 phenotypes need to be developed.

MSC2s have been used for the treatment of a variety of immune-related nephropathies and have been shown to be safe and effective. However, most have only been validated in animal models; clinical trials are lacking. Therefore, it is necessary to conduct more in-depth mechanistic studies on enhancing the immunosuppressive function of MSCs to better define MSCs and their subpopulations, especially MSC2s. The ultimate goal is to develop an internationally recognized standard for the definition of the MSC1/MSC2 phenotypes to facilitate the clinical application of MSCs.

## Author contributions

Both authors listed have made a substantial, direct, and intellectual contribution to the work and approved it for publication.

## Funding

This study was supported by a grant (No. 2020YFA0113004) from the National Key Research and Development Program of China and a grant (No. 81830060) from the National Natural Science Foundation of China.

## Conflict of interest

The authors declare that the research was conducted in the absence of any commercial or financial relationships that could be construed as a potential conflict of interest.

## Publisher's note

All claims expressed in this article are solely those of the authors and do not necessarily represent those of their affiliated organizations, or those of the publisher, the editors and the reviewers. Any product that may be evaluated in this article, or claim that may be made by its manufacturer, is not guaranteed or endorsed by the publisher.
